# Functional impact of long COVID among healthcare workers with comorbidities in Quebec, Canada: a cross-sectional study

**DOI:** 10.1136/bmjph-2025-004108

**Published:** 2026-02-17

**Authors:** Sabine Isangwe, Denis Talbot, Marie-France Coutu, Elisabeth Canitrot, Simon Décary, Emilia Liana Falcone, Manale Ouakki, Philippe Latouche, Alain Piché, Marc Simard, Marianne Balem, Gaston De Serres, Sara Carazo

**Affiliations:** 1 Department of Social and Preventive Medicine, Laval University, Québec City, Quebec, Canada; 2 Centre de recherche du CHUQ, Québec City, Quebec, Canada; 3 Centre de recherche Charles-Le Moyne, Montreal, Quebec, Canada; 4 Direction de la santé environnementale, Quebec National Institute of Public Health, Québec City, Quebec, Canada; 5 Faculty de Medicine and Health Sciences, University of Sherbrooke, Sherbrooke, Quebec, Canada; 6 Centre de recherche du CHUS, CIUSSS de l’Estrie CHUS, Sherbrooke, Quebec, Canada; 7 Center for Immunity, Inflammation and Infectious Diseases, Montreal Clinical Research Institute (IRCM), Montreal, Quebec, Canada; 8 Department of Medicine, University of Montreal, Montreal, Quebec, Canada; 9 Quebec National Institute of Public Health, Québec City, Quebec, Canada; 10 The Charles-Le Moyne Research Center, Montreal, Quebec, Canada; 11 School of Rehabilitation, University of Sherbrooke -Longueuil Campus, Montreal, Quebec, Canada

**Keywords:** Health Personnel, Comorbidity, Cross-Sectional Studies

## Abstract

**Introduction:**

Long COVID is a frequent post-infectious chronic condition that impacts quality of life and work performance. Whether individuals with comorbidities experience a greater functional impact of long COVID is unknown. We evaluated the functional impact of long COVID among healthcare workers (HCWs) with chronic cardiovascular diseases, chronic respiratory diseases, obesity or a history of depression, and compared it with that of HCWs without comorbidities.

**Methods:**

We conducted a cross-sectional study in Quebec, Canada. We compared self-reported long COVID cases to COVID-19-infected controls without long COVID on work ability, work functioning, health-related absenteeism, dyspnoea-associated impairment and psychological distress among HCWs (a) with at least one of the four comorbidities, (b) with each of the four comorbidities and (c) without comorbidities. We used inverse probability of exposure and robust Poisson regressions to estimate adjusted prevalence differences (aPD) and prevalence ratios. Comorbidity data were obtained from the Quebec integrated chronic disease surveillance system.

**Results:**

A total of 3754 and 8439 HCWs with and without comorbidities, respectively, were included. Among HCWs with at least one of the four comorbidities, long COVID was associated with higher prevalence of low work ability (aPD=15%, 95% CI: 12% to 18%), low work functioning (aPD=27%, 95% CI: 22% to 31%), health-related long-term absenteeism (aPD=8%, 95% CI: 5% to 11%), dyspnoea-associated impairment (aPD=23%, 95% CI: 19% to 26%) and psychological distress (aPD=24%, 95% CI: 20% to 28%). aPDs were greater among HCWs with comorbidities than among those without for low work ability (p=0.013 for interaction), for low work functioning (p=0.034) and for dyspnoea-associated impairment (p<0.001).

**Conclusion:**

Long COVID is associated with significant functional impairment among HCWs with pre-existing chronic conditions. HCWs with at least one of the four comorbidities experience lower work ability, lower work functioning and more dyspnoea-associated impairment compared with those without comorbidities.

**WHAT IS ALREADY KNOWN ON THIS TOPIC**

- People with comorbidities and those with long COVID both have affected work performance.

**WHAT THIS STUDY ADDS**

- Long COVID is associated with a greater prevalence of low work ability, low work functioning and dyspnoea-associated impairment among workers with existing comorbidities than among those without.

**HOW THIS STUDY MIGHT AFFECT RESEARCH, PRACTICE OR POLICY**

- Public health, employers and physicians should give particular attention to the specific needs of individuals affected by long COVID who already have comorbidities. There is a need for targeted occupational health policies to reduce the functional impact of long COVID among workers with comorbidities.

## Introduction

 Long COVID, or post-COVID-19 condition, is defined as a post-infection chronic condition that occurs after SARS-CoV-2 infection and is present for at least 3 months as a continuous, relapsing and remitting, or progressive disease state that affects one or more organ systems.^1^ Long COVID symptoms may persist for more than 2 years postinfection and affect patients with asymptomatic infection, mild acute COVID-19, as well as those hospitalised during their illness.^2 3^

According to the World Health Organisation, 10%–20% of individuals who have experienced COVID-19 may develop long COVID.^4^ In Canada, a population survey in 2022 reported a 19% risk of long COVID among adults who had COVID-19,^5^ while a population-based survey in Quebec reported a risk of 15% among healthcare workers (HCWs).^6^

Long COVID affects the overall quality of life and work capacity. Studies have described the need for work accommodations or incapacity to resume work 6 months after COVID-19 hospitalisation due to ongoing health issues.^7^ Comorbidities, such as immunosuppression, chronic obstructive pulmonary disease, asthma, ischaemic heart disease, anxiety and/or history of depression and obesity, are host-related risk factors for long COVID.^8^ Comorbidities may also contribute to work impairment, leading to loss of productivity and increased absenteeism, with a greater number of comorbidities being associated with longer sick leave.^9 10^ Both long COVID and comorbidities are associated with substantial functional limitations. However, evidence regarding the specific impact of long COVID on work and daily activities among individuals with comorbidities remains scarce. Research is needed to clarify how long COVID affects individuals with comorbidities and whether this impact differs from that experienced by those without prior comorbid conditions.

Using data from a survey among HCWs in Quebec, Canada, this study aims to evaluate the association between long COVID and work ability, work functioning, health-related absenteeism, dyspnoea-associated impairment and psychological distress among HCWs with chronic cardiovascular diseases, chronic respiratory diseases, obesity or history of depression and to compare these associations with those observed among HCWs without comorbidities.

## Materials and methods

### Study design and population

We conducted a cross-sectional study, based on the 2023 survey conducted by the Quebec National Institute of Public Health among HCWs.^6^ All HCWs aged 18 years and older and residing in the province of Quebec were invited to participate in the survey. This study included participants who had self-reported COVID-19 episodes and belonged to one of the following five subpopulations based on pre-existing comorbidities: chronic cardiovascular diseases (ie, cardiac arrhythmias, congestive heart failure and valvular disease), chronic respiratory diseases (ie, chronic pulmonary disease and respiratory diseases), obesity, history of depression or no known comorbidity. Comorbidities were chosen based on their known association with long COVID and their prevalence in our population.^11 12^ HCWs with a history of long COVID that had resolved by the time of the survey were excluded because the outcomes were related to the situation at the survey’s completion date. Participants with uncertain or inconsistent information regarding the onset and duration of post-COVID-19 symptoms were also excluded. Additional exclusions were students, interns and individuals with incomplete surveys.

### Patient involvement

Long COVID patients were involved in establishing the research question and in the interpretation of the results.

### Data collection

In spring 2023, we collected demographic information, retrospective data on participants’ COVID-19 episodes since March 2020 and symptoms during the acute phase, and prevalent data on persistent post-COVID-19 symptoms and their severity at the time of the survey, using a self-administered questionnaire. The questionnaire also included validated scales to assess work ability, work functioning, health-related absenteeism, dyspnoea-associated impairment and psychological distress.

Data on participants’ comorbidities were obtained from the Quebec integrated chronic disease surveillance system (QICDSS), an administrative database with diagnoses available up to 31 March 2021.^13^ We defined individuals without comorbidities as those having none of the 30 comorbidities identified by QICDSS (see online supplemental table 1) for details.^14^

### Exposition and outcomes

Long COVID cases were defined as HCWs reporting at least one symptom of any severity lasting for at least 12 weeks following an acute episode of COVID-19 and attributed to COVID-19 by the participant. Prevalent cases were those with long COVID symptoms present at survey completion date. COVID controls were HCWs reporting at least one episode of COVID-19 whose symptoms lasted less than 12 weeks. Long COVID severity among prevalent cases was categorised according to the self-reported severity of post-COVID-19 symptoms as follows: mild if only mild symptoms were reported, moderate if one or more moderate but no severe symptoms were reported and severe if one or more severe, persistent symptoms were reported.

Work ability and absenteeism were assessed using four items from the Work Ability Index (WAI) among HCWs with paid employment at the time of the survey^15^ (online supplemental table 2). Questions were asked about self-perceived work ability before the pandemic and at the time of the survey. Low work ability was evaluated globally (score of <6 out of 10) and in relation to physical or mental job demands (rather poor or poor, corresponding to a score of <3 out of 5). For absenteeism, a question was asked about the number of full days the participants were absent from work due to health-related issues during the last year. Long-term absenteeism was defined as 100 or more workdays missed, consecutively or not, during the last year due to health-related issues. The 100-day threshold corresponds to the largest category in the validated WAI’s question.^15^

Work functioning was evaluated using the Work Role Functioning Questionnaire V.1.2 (WRFQ).^16^ The WRFQ measures the perceived difficulty for the worker to meet job demands based on an individual’s health condition. It is a 27-item instrument covering five subscales: work schedule demands, work output demands, physical demands, mental/social demands and flexibility demands, assessed among HCWs with paid employment during the 4 weeks prior to the survey (online supplemental table 3). Participants rated their difficulty over the past 4 weeks on a five-point scale (from ‘difficult all the time’ to ‘never difficult’). Subscale scores from 0% to 100% were calculated by averaging item scores and multiplying by 25, indicating the proportion of time the worker was able to meet job demands. We defined low work functioning by a total WRFQ score of ≤75%, the mean score among all Quebec survey participants.^17^

Dyspnoea-associated impairment was evaluated using the Modified Medical Research Council scale^18 19^ (online supplemental table 4). This is a commonly used scale for evaluating the degree of impairment that dyspnoea poses in patients with chronic respiratory diseases, from none (grade 0) to near-total incapacity (grade 4). Dyspnoea-associated impairment was defined as a score of >1 out of 4. Participants were asked to report their dyspnoea-associated impairment before the pandemic and at the time of the survey.

Psychological distress was assessed using the Kessler K6 scale^20 21^ (online supplemental table 5). This is a 6-question scale that evaluates the frequency (‘never’ to ‘all the time’) of feelings such as nervousness, hopelessness, restlessness, feeling depressed, worthlessness and effortfulness over the preceding month. Out of 24 points, scores were categorised as follows: absence of high psychological distress (score <7), high psychological distress (score of 7–12) and very high psychological distress (score ≥13).^22^ An additional question evaluated whether the psychological distress was completely or partially associated with persistent COVID-19 symptoms.

### Statistical analysis

The minimal detectable difference was calculated assuming 80% statistical power and a two-sided significance level of 5%. Among HCWs with at least one of the four comorbidities, our study population allowed detection of a prevalence difference of 3.3% for low work ability, 5.8% for low work functioning, 4.1% for health-related absenteeism, 4.2% for dyspnoea-associated impairment and 6.8% for high psychological distress. Among HCWs without comorbidities, the respective minimal detectable differences were 1.9%, 4.2%, 2.2%, 2.1% and 5.3%. This indicates that the study was well powered to detect small to moderate differences depending on the outcome and comorbidity status.

Baseline characteristics of the population were summarised using means and SDs for continuous variables and frequencies and percentages for categorical variables.

The principal analysis compared the risk of each outcome among long COVID cases compared with COVID controls for each subpopulation of defined comorbidities or HWCs without comorbidities using inverse probability of exposure weighting to estimate the unadjusted and adjusted prevalence differences (aPDs) along with their 95% CIs. For comparability with the literature, we also used the robust Poisson regressions to estimate the unadjusted and adjusted prevalence ratios (aPRs) along with their 95% CIs. The robust Poisson regression allows direct estimation of the PRs, compared with logistic regression, which estimates the ORs (ORs), which may overestimate PRs when the outcome is common. It is also less prone to non-convergence than a log-binomial model.^23^ Measures of association were compared between those with and without comorbidities by introducing an interaction term between long COVID and the presence of any comorbidity. Multivariate models were adjusted for the following potential confounding factors: age (18–44, 45–54 and ≥55 years), sex, number of comorbidities, ethnicity (White vs non-White), workplace (acute-care hospital; long-term care facility and private homes for older people; community clinics, family medicine groups and other clinics; rehabilitation centre and other settings), type of work (nursing staff and physicians; personal support workers and healthcare assistants; administrative and management staff and other professionals) and the social and material deprivation indexes (five quintiles).^24^

The following secondary analyses were also conducted: (1) restricting to moderate or severe long COVID cases; (2) for each subscale of the WRFQ; (3) for work ability in relation to physical and mental job demands; (4) for very high psychological distress. Statistical analyses were performed using SAS V.9.4.

## Results

### Study population

**Figure 1:**
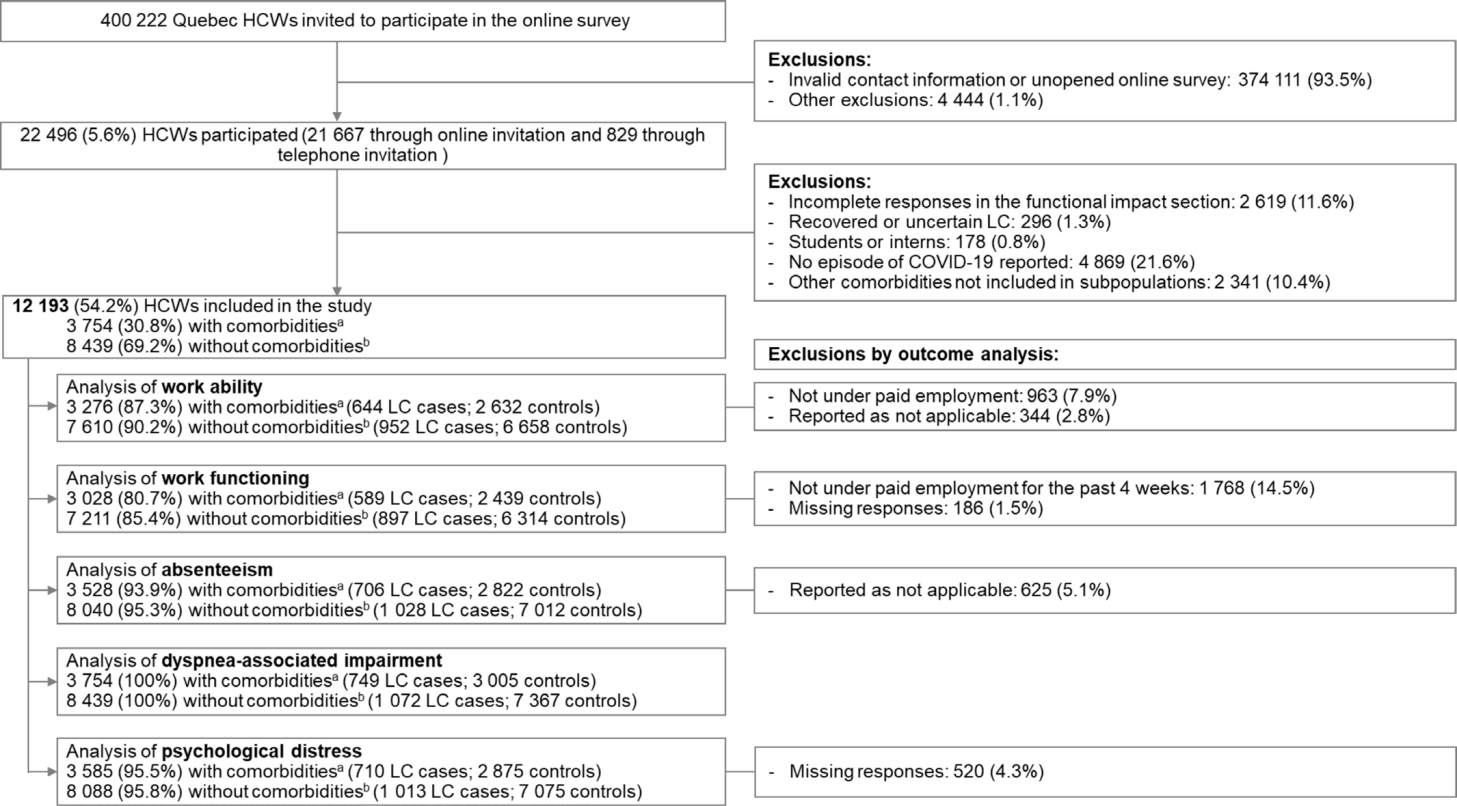
Flowchart of study population for each outcome. ^a^Healthcare workers with at least one of the four comorbidities: chronic cardiovascular diseases, chronic respiratory diseases, obesity and a history of depression. HCWs, healthcare workers; LC, long COVID. ^b^Healthcare workers without comorbidities according to the Quebec integrated chronic disease surveillance system, which provides information on the following comorbidities: AIDS/HIV, alcohol abuse, anaemia, cancer, cardiovascular disease, cerebrovascular disease, coagulopathy, dementia, depression, drug abuse, fluid and electrolyte disorders, hepatic disease, hypertension, hypothyroidism, immune system problems, neurological disorders, obesity, paralysis, peripheral vascular disorders, psychosis, pulmonary circulation disease, renal disease, rheumatoid arthritis, ulcer disease, weight loss, chronic pulmonary disease and respiratory disease.

Of 400 222 HCWs invited, 22 496 (5.6%) completed the questionnaire (figure 1). As reported elsewhere,^6^ sociodemographic characteristics such as sex, region of residence and comorbidities were similar between participants and overall HCW population. Participants were older than the target population, with 65.8% aged 40 years or older compared with 55.0% in the target group. Participants were excluded if they had recovered from long COVID (n=296, 1.3%), students (n=178, 0.8%), reported no known COVID-19 infection (n=4 869, 21.6%) or had comorbidities not considered in this study (n=2 341, 10.4%). HCWs were excluded for each outcome if not applicable (eg, not working at the time of the survey or missing data).

Among participants with comorbidities, 749 (19.9%) had long COVID, and 3 005 (80.0%) were COVID controls (table 1). Among those without comorbidities, 1 072 (12.7%) had long COVID. The study population was mostly female, White, with an average age of 45.9 and 43.5 years for participants with and without comorbidities, respectively. HCWs with comorbidities had an average of two comorbidities among those measured (online supplemental table 1). Nursing was the most prevalent occupation, and acute-care hospitals were the most common workplace.

**Table 1:** Characteristics of long COVID cases and COVID controls by comorbid status

	HCWs with comorbidities			HCWs without comorbidities		
	Long COVID cases	COVID controls	Total	Long COVID cases	COVID controls	Total
N (line %)	749 (19.9)	3005 (80.0)	3754	1072 (12.7)	7367 (87.3)	8439
Characteristics	N (%)	N (%)	N (%)	N (%)	N (%)	N (%)
Sex						
Female	652 (87.1)	2612 (86.9)	3264 (86.9)	900 (83.9)	5857 (79.5)	6757 (80.1)
Male	97 (12.9)	393 (13.1)	490 (13.1)	172 (16.0)	1510 (20.5)	1682 (19.9)
Age (years)						
Mean±SD	47.3±10.7	45.6±11.6	45.9±11.5	44.7±10.9	43.3±11.9	43.5±11.8
18–44	307 (41)	1534 (51.1)	1841 (49.04)	567 (52.9)	4243 (57.6)	4810 (57)
45–54	241 (32.2)	764 (25.4)	1005 (26.8)	289 (26.9)	1687 (22.9)	1976 (23.4)
≥55	201 (26.8)	707 (23.5)	908 (24.2)	216 (20.1)	1437 (19.5)	1653 (19.6)
Comorbidities*						
Mean number±SD	1.9±1.3	1.8±1.2	1.9±1.2	NA	NA	NA
Chronic cardiovascular diseases	119 (15.9)	489 (16.3)	608 (16.2)	NA	NA	NA
Chronic respiratory diseases	257 (34.3)	947 (31.5)	1204 (32.1)	NA	NA	NA
Obesity	160 (21.4)	596 (19.8)	756 (20.1)	NA	NA	NA
History of depression	412 (55.0)	1561 (51.0)	1973 (52.6)	NA	NA	NA
More than one comorbidity	391 (52.2)	1407 (46.8)	1798 (47.9)	NA	NA	NA
Race/ethnicity						
White	661 (88.3)	2771 (92.2)	3432 (91.4)	893 (83.3)	6358 (86.3)	7251 (85.9)
Black	21 (2.8)	67 (2.2)	88 (2.3)	50 (4.7)	265 (3.6)	315 (3.7)
Hispanic	13 (1.7)	41 (1.4)	54 (1.4)	35 (3.3)	137 (1.9)	172 (2)
Arab	9 (1.2)	25 (0.8)	34 (0.9)	21 (1.9)	125 (1.7)	146 (1.7)
Asian	6 (0.8)	28 (0.93)	34 (0.9)	24 (2.2)	242 (3.3)	266 (3.2)
Indigenous	12 (1.6)	18 (0.6)	30 (0.8)	7 (0.7)	26 (0.4)	33 (0.4)
Other categories	27 (3.6)	55 (1.8)	82 (2.2)	42 (3.9)	214 (2.9)	256 (3)
Occupation						
Nursing staff	202 (26.9)	851 (28.3)	1053 (28.1)	322 (30.0)	1756 (23.8)	2078 (24.6)
Administrative and management staff	147 (19.6)	607 (20.2)	754 (20.1)	181 (16.9)	1407 (19.1)	1588 (18.8)
Psychosocial workers	79 (10.6)	366 (12.2)	445 (11.9)	110 (10.3)	725 (9.8)	835 (9.9)
Support and healthcare assistants	102 (13.6)	207 (6.9)	309 (8.2)	130 (12.1)	611 (8.3)	741 (8.8)
Physicians	22 (2.9)	113 (3.8)	135 (3.6)	40 (3.7)	497 (6.7)	537 (6.4)
Other professionals	171 (22.8)	800 (26.6)	971 (25.9)	289 (26.9)	2371 (32.2)	2660 (31.5)
Workplace						
Acute-care hospitals	298 (39.8)	1290 (42.9)	1588 (42.3)	455 (42.4)	3278 (44.5)	3733 (44.2)
Clinics and family medicine groups	121 (16.2)	509 (16.9)	630 (16.8)	151 (14.1)	1215 (16.5)	1366 (16.2)
Long-term care facility	139 (18.6)	337 (11.2)	476 (12.7)	173 (16.1)	741 (10.1)	914 (10.8)
Rehabilitation centre	42 (5.6)	204 (6.8)	246 (6.6)	77 (7.2)	550 (7.5)	627 (7.4)
Remote work	26 (3.5)	161 (5.4)	187 (4.9)	59 (5.5)	395 (5.4)	454 (5.4)
Home care	23 (3.1)	89 (2.9)	112 (2.9)	28 (2.6)	153 (2.1)	181 (2.1)
Private homes for older people	11 (1.5)	29 (0.9)	40 (1.1)	12 (1.1)	48 (0.7)	60 (0.7)
Other settings	89 (11.9)	386 (12.9)	475 (12.7)	117 (10.9)	987 (13.4)	1104 (13.1)
Material deprivation index†						
Unmatched‡	45 (6.0)	109 (3.6)	154 (4.1)	38 (3.5)	272 (3.7)	310 (3.7)
Quintile 1	139 (18.6)	630 (20.9)	769 (20.5)	232 (21.6)	1958 (26.6)	2190 (25.9)
Quintiles 2–4	453 (60.5)	1884 (62.7)	2337 (62.1)	652 (60.8)	4284 (58.2)	4936 (58.5)
Quintile 5	112 (14.9)	382 (12.7)	494 (13.2)	150 (13.9)	853 (11.6)	1003 (11.9)
Social deprivation index†						
Unmatched‡	45 (6.0)	109 (3.6)	154 (4.1)	38 (3.5)	272 (3.7)	310 (3.7)
Quintile 1	135 (18.0)	600 (19.9)	735 (19.6)	202 (18.8)	1445 (19.6)	1647 (19.5)
Quintiles 2–4	452 (60.3)	1773 (59.0)	2225 (59.3)	625 (58.3)	4290 (58.2)	4915 (58.2)
Quintile 5	117 (15.6)	523 (17.4)	640 (17.1)	207 (19.3)	1360 (18.5)	1567 (18.6)

* Number of comorbidities among the following: AIDS/HIV, alcohol abuse, anaemia, cancer, cardiovascular disease, cerebrovascular disease, coagulopathy, dementia, depression, drug abuse, fluid and electrolyte disorders, hepatic disease, hypertension, hypothyroidism, immune system problems, neurological disorders, obesity, paralysis, peripheral vascular disorders, psychosis, pulmonary circulation disease, renal disease, rheumatoid arthritis, ulcer disease, weight loss, chronic pulmonary disease and respiratory disease.

† Quintile 1, very advantaged; quintiles 2–4, neutral (middle range); quintile 5, very disadvantaged.

‡ Unmatched: include unpaired individuals due to invalid or erroneous postal codes.

.HCWs, healthcare workers; NA, not applicable .

### Long COVID impact on work ability, absenteeism and work functioning

Among COVID controls with comorbidities, 4.7% reported low work ability, 24.7% low work functioning and 8.1% long-term absenteeism, while controls without comorbidities reported, respectively, a prevalence of 2.8%, 21.6% and 3.1% for each outcome (online supplemental figure 1). Independent of the comorbidity status, HCWs reported having good work ability before the pandemic (less than 1.5% reported low work ability) (online supplemental figure 2).

**Figure 2:**
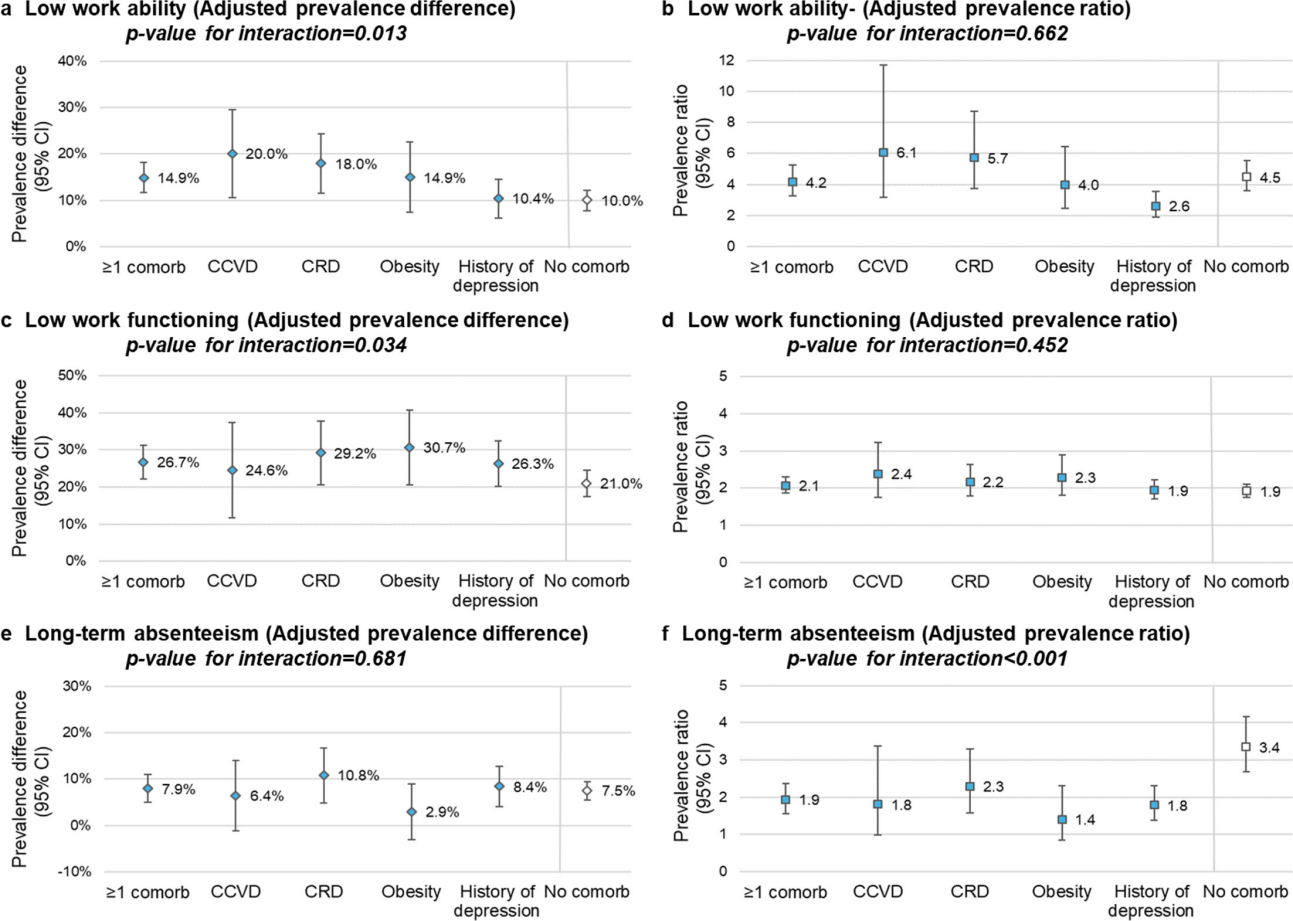
Adjusted prevalence difference and ratio of low work ability, low work functioning and long-term absenteeism among healthcare workers with long COVID. (a) low work ability adjusted prevalence difference; (b) low work ability adjusted prevalence ratio; (c) low work functioning adjusted prevalence difference; (d) low work functioning adjusted prevalence ratio; (e) long-term absenteeism adjusted prevalence difference; (f) long-term absenteeism adjusted prevalence ratio. CCVD, chronic cardiovascular disease; Comorb, comorbidity; CRD, chronic respiratory disease. Note 1: models adjusted for age, sex, occupation, workplace, number of comorbidities, social deprivation index and material deprivation index. Note 2: the reported p-values represent the interaction effects of long COVID and the presence or absence of comorbidities on different functional impacts.

Among HCWs with comorbidities, long COVID was associated with a 14.9% higher prevalence of low work ability (95% CI: 11.6% to 18.2%), 26.7% higher prevalence of low work functioning (95% CI: 22.1% to 31.3%) and 7.9% higher prevalence of long-term absenteeism (95% CI: 4.9% to 10.9%) (figure 2, online supplemental table 6). The absolute increase of low work ability and low work functioning was higher among HCWs with comorbidities than those without (p interaction=0.013 and 0.034, respectively) (figure 2). The association between long COVID and low work ability was particularly important among HCWs with chronic cardiovascular (aPD=20.0% (95% CI: 10.6% to 29.5%)) and respiratory diseases (aPD=17.9% (95% CI: 11.6% to 24.3%)). The aPRs (measuring the relative impact) were comparable between those with or without comorbidities for low work ability (aPR=4.2 vs 4.5) and for low work functioning (aPR=2.1 vs aPR=1.9).

While the estimated absolute impact of long COVID on absenteeism was comparable among those with and without comorbidities (aPD=7.9% vs aPD=7.5%, respectively), the estimated relative impact was greater among those without comorbidities (aPR=3.4 (95% CI: 2.7 to 4.2)) than among those with comorbidities (aPR=1.9 (95% CI: 1.6 to 2.4)), with a p-value of interaction<0.001 (figure 2, online supplemental table 6).

In secondary analyses restricted to moderate or severe long COVID, the estimated absolute impact on low work ability, low work functioning and long-term absenteeism was greater (aPD of 20.1%, 32.9% and 10.1%, respectively) (online supplemental table 7). Long COVID estimated impact on physical job demands was greater than on mental job demands in all subpopulations (eg, aPD among those with chronic cardiovascular diseases of 21.0% for physical vs 15.5% for mental job demands) (online supplemental table 8). Work schedule demand was the most affected of the work functioning subscales, with 85.6% prevalence among those with long COVID and 71.9% among those without (online supplemental table 9).

### Long COVID impact on dyspnoea-associated impairment

Among COVID controls, dyspnoea-associated impairment was more prevalent among HCWs with comorbidities compared with those without (6.5% vs 2.3%), particularly among those with obesity (9.7%) (online supplemental figure 1). Long COVID cases reported a lower proportion of dyspnoea-associated impairment before the pandemic than at the time of the survey (0.5% vs 16.4% for HCWs without comorbidity, 2.8% vs 30.9% for HCWs with comorbidities and 5.0% vs 43.1% for HCWs with obesity) (online supplemental figure 2 and table 6).

**Figure 3:**
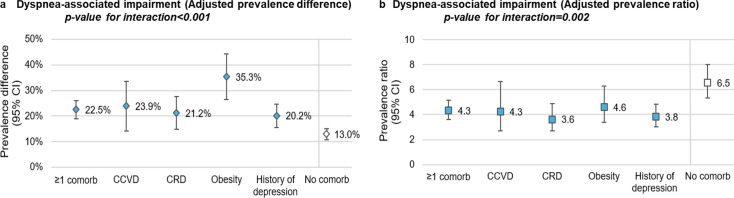
Adjusted prevalence difference and ratio for dyspnoea-associated impairment among healthcare workers with long COVID. (a) dyspnoea-associated impairment adjusted prevalence; (b) dyspnoea-associated impairment adjusted prevalence ratio. CCVD, chronic cardiovascular disease; Comorb, comorbidity; CRD, chronic respiratory disease. Note 1: adjusted for age, sex, occupation, workplace, number of comorbidities, material deprivation index and social deprivation index. Note 2: the reported p-values represent the interaction effects of long COVID and the presence or absence of comorbidities on dyspnoea-associated impairment.

The estimated absolute impact of long COVID on dyspnoea-associated impairment was greater among those with comorbidities (aPD=22.5% (95% CI: 19.0% to 26.0%)) than among those without comorbidities (aPD=13.0% (95% CI: 10.8% to 15.2%), p interaction<0.001) (figure 3, online supplemental table 6). HCWs with long COVID were between 3.6 and 6.5 times more likely, depending on the comorbidity subpopulation, to report dyspnoea-associated impairment than those without it. Moreover, HCWs with obesity were the most affected by dyspnoea-associated impairment in both relative (aPR=4.6) and absolute (aPD=35.3%) measures.

When restricted to moderate or severe long COVID, the estimated impact on dyspnoea-associated impairment was greater than for overall long COVID cases (online supplemental table 7).

### Long COVID impact on psychological distress

Among COVID controls, 44.6% and 34.9% of HCWs with and without comorbidities reported high psychological distress at the time of the survey (online supplemental figure 3). Among long COVID cases with comorbidities, 69.1% had high psychological distress (31.9% very high psychological distress), of which 20.6% and 35.8% reported that it was fully or partially linked with their post-COVID-19 symptoms (online supplemental figure 3 and table 10).

**Figure 4:**
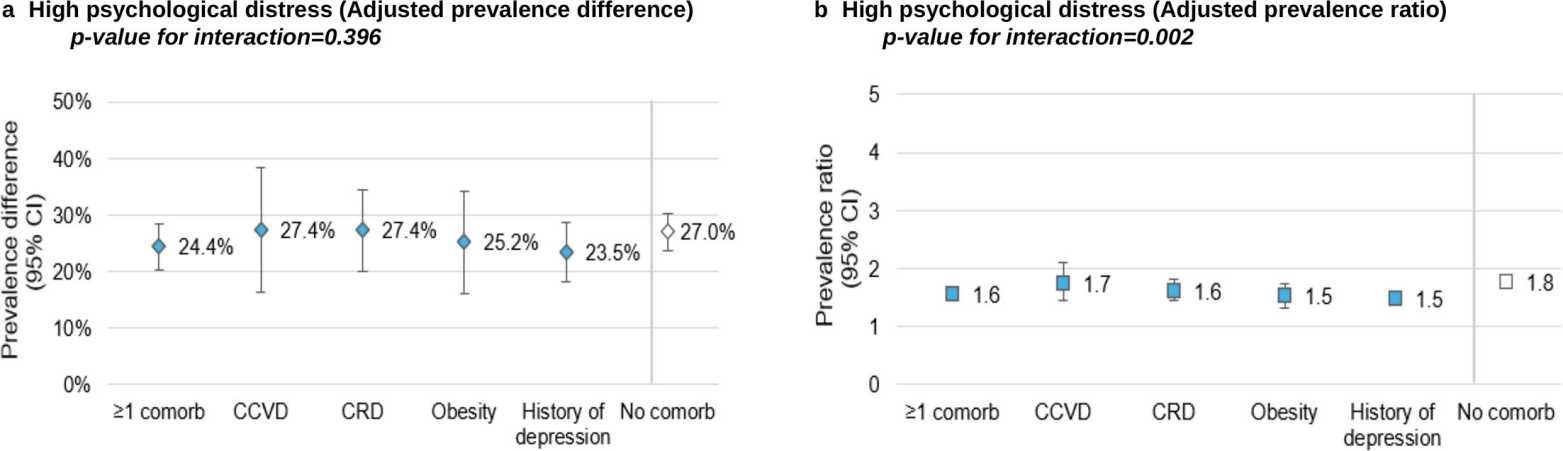
Adjusted prevalence difference and ratio for high psychological distress among healthcare workers with long COVID. (a) high psychological distress adjusted prevalence difference; (b) high psychological distress adjusted prevalence ratio. CCVD, chronic cardiovascular disease; Comorb, comorbidity; CRD, chronic respiratory disease. Note 1: adjusted for age, sex, occupation, workplace, number of comorbidities, material deprivation index and social deprivation index. Note 2: the reported p-values represent the interaction effects of long COVID and the presence or absence of comorbidities on high psychological distress.

The absolute increase in prevalence of high psychological distress associated with long COVID was similar among HCWs without comorbidities (aPD=27.0% (95% CI: 23.7% to 30.3%)) compared with those with at least one of the comorbidities (aPD=24.4% (95% CI: 20.4% to 28.4%), p interaction=0.396) (figure 4, online supplemental table 6). The relative prevalence increase was greater among HCWs without comorbidities (aPR=1.8 (95% CI: 1.7 to 1.9)) than among HCWs with comorbidities (aPR=1.6 (95% CI: 1.5 to 1.7), p interaction=0.002) (figure 4, online supplemental table 6).

## Discussion

Our study shows that long COVID is associated with low work ability, low work functioning, health-related absenteeism, dyspnoea-associated impairment and high psychological distress among HCWs with comorbidities. HCWs with moderate or severe long COVID experienced more functional impairment. When comparing HCWs with comorbidities to those without, we observed greater prevalence differences for low work ability and low work functioning but similar rates for long-term absenteeism. Conversely, the prevalence ratios were similar for low work ability and low work functioning and lower for long-term absenteeism, underscoring the potential underestimation of long COVID impact when using relative measures in populations who already have a baseline impact (among controls) due to comorbidities.

Previous studies have shown that the presence of comorbidities is associated with long COVID risk.^8 25^ Moreover, the presence of chronic diseases, including mental diseases, obesity, cardiac diseases and respiratory diseases, is associated with an increase in absenteeism and a decrease in work productivity and ability.^9 10 26 27^ Similar results were obtained in our study, where controls (participants without long COVID) with comorbidities had a greater prevalence of low work ability and long-term absenteeism than those without comorbidities.

Previous studies have reported on the impact of long COVID on work ability and absenteeism.^2528^,^30^ A German study found that health and social workers reported having a very good work ability (score above 9 out of 10) before the pandemic, while in 2021, participants with long COVID reported a lower average score of work ability (6.8 vs 8.9, p value<0.01).^25^ A study from the UK among employees who had had COVID-19, among whom 79% had long COVID, reported that 38% rated their physical work ability as rather poor or very poor, and 33% described their mental work ability as rather poor or very poor.^28^ A study conducted in Thailand observed that nursing personnel with long COVID reported lower work ability than those without (adjusted OR (aOR)=3.6 (95% CI 1.6 to 8.3)).^29^ Additionally, the presence of chronic diseases was associated with lower work ability in the long COVID group (aOR=2.9 (95% CI 1.2 to 6.9)). We have not found studies that specifically evaluated the impact of long COVID in individuals with comorbidities. Our findings are in line with the literature, showing a high work ability before the pandemic and a decrease in work ability among long COVID cases at the time of the survey. Importantly, this study is the first to assess the work-related impact of long COVID among patients with common comorbidities. Furthermore, we found that the absolute increase in prevalence of low work ability among HCWs with long COVID was greater among those with at least one of the comorbidities than in those without comorbidities. A nationwide Canadian survey found that 22.3% of long COVID cases missed work or school due to long COVID, with an average of 24 days of absences due to health issues.^5^ In our study, long COVID was associated with health-related absenteeism of ≥100 days in the previous year, both among participants with comorbidities (aPD=7.9%) and without comorbidities (aPD=7.5%).

Some comorbidities, as well as long COVID, are associated with limitations in physical activities.^31^,^34^ A retrospective study of 156 patients followed in a long COVID clinic in New York showed that 40% had moderate to severe disability due to dyspnoea and a reduced frequency of physical activities since their COVID-19 infection.^34^ Among those patients, more than half had pre-existing chronic diseases, but the results were not stratified by comorbidity status. We found that long COVID was associated with four times greater prevalence of dyspnoea-associated impairment among participants with comorbidities. HCWs with obesity were the most affected by dyspnoea-associated impairment at baseline (10% of HCWs without long COVID) and had the highest increase in dyspnoea-associated impairment when suffering from long COVID.

According to a 2021 survey conducted in Quebec, 50.7% of HCWs experienced high psychological distress, among which 81.5% considered their psychological distress as work-related.^35^ Psychological distress, however, did not seem to be related to SARS-CoV-2 infection. In 2023, we still observed a high prevalence (44.6%) of psychological distress among HCWs without long COVID. Long COVID was associated with approximately 25% higher prevalence of psychological distress, indicating a substantial psychological burden in this population. While some comorbidities (ie, history of depression, obesity and chronic respiratory disease) were associated with higher psychological distress at baseline, the estimated absolute and relative impact of long COVID did not change with comorbidity status. A study in the USA showed that young adults with long COVID experienced very high psychological distress more often than those without (aOR=1.5 (99% CI 1.4 to 1.7)).^36^ Similarly, we observed that very high psychological distress was twice as prevalent among HCWs with long COVID, showing its impact on mental health in a population already touched by the pandemic.

Our study has strengths and limitations. HCWs were recruited through a population-based survey, which allowed us to include HCWs with various occupations and from different workplaces and backgrounds. However, the participation rate was low (5.6%), probably because of the length of the 30 min questionnaire. Besides, only complete surveys were used, which has the potential to introduce selection bias. A short telephonic survey conducted to evaluate the representativeness of the participants suggested that long COVID cases were more likely to respond to the long online survey.^6^ This would not introduce bias to our study, provided that the participating long COVID cases adequately represent the full spectrum of the disease within the source population. However, those with severe functional impacts might be under-represented due to participants’ limited ability to complete a long questionnaire, which can lead to underestimation of long COVID impact or, conversely, might be more interested in participating, leading to overestimation. We used self-reported persisting symptoms at the time of the survey to minimise recall bias when identifying long COVID cases, but participants were not evaluated by healthcare professionals to exclude other medical conditions that could explain the symptoms.^37^ If individuals without long COVID but with symptoms related to other conditions are defined as long COVID cases, it would lead to differential misclassification. On the other hand, the excluded cases who had recovered from long COVID were likely less severe than prevalent cases.^6^ While this exclusion may lead to a higher estimated functional impact of long COVID in our study, the findings reflect the situation of more persisting chronic cases, which carry the greatest public health burden. The large sample size allowed us to perform analyses stratified according to four specific comorbidities. However, in our working-age population, the prevalence of comorbidities was not high, limiting our capacity to evaluate other comorbidities that might modify the functional impact of long COVID. We used validated tools to measure our outcomes, but only some of the questions of the WAI were used, limiting comparability with other studies. Given that the outcomes relied on self-reported questionnaires, they are susceptible to information bias due to subjective reporting or inaccurate recall. Recall bias should, however, be limited as questions were related to the moment of the questionnaire or the previous weeks. Another limitation is the cross-sectional design, which limits the ability to establish causality. However, we tried to ensure the temporality between long COVID and outcomes by evaluating some of them pre-pandemic and at the time of the survey, which showed the similarity of long COVID cases and controls before the pandemic. We also used data from QICDSS to identify HCWs with comorbidities, which gave us access to a large sample of individuals with various comorbidities and ensured that comorbidities were diagnosed prior to or early during the pandemic. Comorbidity status was intended to represent pre-existing vulnerability rather than conditions arising after COVID-19 infection. Therefore, using comorbidities measured before or early in the pandemic (before the Omicron wave) minimises the risk of reverse causation (eg, comorbidities developed as a consequence of COVID-19). However, the QICDSS is not exhaustive and depends on healthcare access and behaviour, and some participants may have developed a comorbidity after data extraction in March 2021, potentially leading to under-ascertainment of some HCWs with comorbidities. Such misclassification is expected to underestimate the differences in the functional impact of long COVID between individuals with comorbidities and those without. However, healthcare access problems should be limited in our population of HCWs. Our analyses were adjusted for multiple important confounding factors, but residual confounding may remain.

Our results have important implications for occupational medicine. They show that HCWs with comorbidities have a particularly important burden when affected by long COVID compared with those without comorbidities, particularly in terms of reduced work ability and work functioning, and dyspnoea-associated impairment. Interventions such as work accommodation, access to rehabilitation programmes, mental health support and gradual work titration should be considered to support workers with long COVID.^38 39^

In conclusion, this study is the first to document the significant functional impairment experienced by HCWs with long COVID and pre-existing comorbidities. Our findings enhance understanding of the disproportionate burden of long COVID among specific subpopulations of HCWs. These results emphasise the need for special considerations and support for these individuals to mitigate further functional impairment. Healthcare systems and employers must consider the unique challenges faced by workers with comorbidities by providing necessary resources and accommodations to ensure their well being and maintain productivity.

## Supplementary material

10.1136/bmjph-2025-004108 online supplemental file 1

## Data Availability

Data may be obtained from a third party and are not publicly available.
