# Supplementary material for: Functional impact of long COVID among healthcare workers with comorbidities in Quebec, Canada: a cross-sectional study
Source: BMJ Public Health. 2026 Feb 17;4(1):e004108. doi: 10.1136/bmjph-2025-004108 (PMC12927349; doi:10.1136/bmjph-2025-004108)
Supplement: online supplemental file 1 [file bmjph-4-1-s001.pdf]

Functional impact of long COVID among healthcare workers with comorbidities in Quebec, Canada.

Authors:

Sabine Isangwe, MSc[1,2]; Denis Talbot, PhD[1,2]; Marie-France Coutu, PhD[3]; Elisabeth Canitrot, PhD[4]; Simon Décary, PhD[5,6]; Emilia Liana Falcone, MD PhD[7,8]; Manale Ouakki, MSc[9]; Philippe Latouche[10]; Alain Piché, MD MSc[5,6]; Marc Simard, PhD[1,9]; Marianne Balem, PhD[3,11]; Gaston De Serres, MD PhD[1]; Sara Carazo, MD PhD[1,2,9]

Affiliations:

[1] Department of Social and Preventive Medicine, Faculty of Medicine, Laval University, Quebec, Canada

[2] The CHU de Québec-Laval University Research Center, Quebec, Canada

[3] The Charles - Le Moyne Research Center (CRCLM) CISSS de la Montérégie-Centre, Longueuil, Canada

[4] Direction de la santé environnementale, au travail et de la toxicologie, Quebec National Institute of Public Health, Quebec, Canada

[5] Faculty de Medicine and Health Sciences, University of Sherbrooke, Quebec, Canada

[6] Centre de recherche du CHUS, CIUSSS de l'Estrie-CHUS, Sherbrooke, Quebec, Canada

[7] Center for Immunity, Inflammation and Infectious Diseases, Montreal Clinical Research Institute (IRCM), Montreal, Quebec, Canada

[8] Department of Medicine, University of Montreal, Montreal, Quebec, Canada

[9] Quebec National Institute of Public Health, Quebec, Canada

[10] Patient-partner

[11] Centre for Action in Work Disability Prevention and Rehabilitation (CAPRIT), School of Rehabilitation, University of Sherbrooke - Longueuil Campus, Longueuil, Quebec, Canada.

Supplementary materials

Supplementary Table 1: List of chronic diseases identified by the Quebec integrated chronic disease surveillance system 3

Supplementary Table 2: Work ability index questions used to evaluate work ability and absenteeism 4

Supplementary Table 3: Work role functioning questionnaire to evaluate work functioning 5

Supplementary Table 4: The Modified medical research council scale to evaluate dyspnea-associated impairment 6

Supplementary Table 5: The Kessler scale K6 to evaluate the psychological distress 7

Supplementary Table 6: Prevalence differences and prevalence ratios of low work-related capacity, dyspnea-associated impairment, and high psychological distress among healthcare workers with long COVID........ 8

Supplementary Table 7: Prevalence differences and prevalence ratios of low work-related capacity, dyspnea-associated impairment, and high psychological distress among healthcare workers with moderate or severe long COVID........ 10

Supplementary Table 8: Prevalence differences and prevalence ratios of low work ability, overall and in relation to physical and mental job demands among healthcare workers with long COVID. 12

Supplementary Table 9: Prevalence differences and prevalence ratios of the five subscales of low work functioning among healthcare workers with long COVID. 14

Supplementary Table 10: Prevalence differences and prevalence ratios of very high psychological distress among healthcare workers with long COVID. 16

Supplementary Figure 1: Prevalence of low work ability, low work functioning, long-term absenteeism, and dyspnea-associated impairment among healthcare workers without long COVID. 17

Supplementary Figure 2: Prevalence of self-reported low work ability and dyspnea-associated impairment of healthcare workers before the pandemic. 18

Supplementary Figure 3: Prevalence of high psychological distress among healthcare workers at the time of the survey and its attribution to the post-COVID-19 symptoms. 19

Supplementary Table 1: List of chronic diseases identified by the Quebec integrated chronic disease surveillance system

The Quebec integrated chronic disease surveillance system (QICDSS) uses data from the following sources: hospitalisation database, medical services, death registry, health insurance registry, and pharmaceutical services database. The diagnostic codes used were based on the International Classification of Diseases, Canadian enhancement of the tenth revision (ICD-10-CA) since April 1st, 2006. To determine whether a person had a specific condition, two medical acts or one hospitalization with the relevant diagnosis had to be recorded within the past 5 years for depression or 10 years for chronic cardiovascular diseases, chronic respiratory disease, and obesity.

Comorbidities identified by Quebec integrated chronic disease surveillance system
AIDS/HIV
Alcohol abuse
Anemia
Cancer
Cardiac arrhythmias
Cerebrovascular disease
Chronic pulmonary disease
Coagulopathy
Congestive heart failure
Dementia
Depression
Diabetes
Drug Abuse
Fluid and electrolyte disorders
Hepatic disease
Hypertension
Hypothyroidism
Immune system problems
Neurological disorders
Obesity
Paralysis
Peripheral vascular disorders
Psychosis
Pulmonary circulation disorders
Respiratory diseases
Renal disease
Rheumatoid arthritis
Ulcer disease
Valvular disease
Weight loss

Abbreviations: AIDS/HIV, Acquired immunodeficiency syndrome/ Human immunodeficiency virus.

Supplementary Table 2: Work ability index questions used to evaluate work ability and absenteeism[1]

Items and questions	Choice of answers and scores	Construction of indicators
**Overall work ability**		It was evaluated before the pandemic and at the time of the survey
How do you rate your overall work ability compared to your highest work ability ever?	2 – poor<br>4 – rather poor<br>6 – fair<br>8 – good<br>10 – very good	Low work ability: score less than 6 out of 10
**Work ability in relation to job demand**		It was evaluated before the pandemic and at the time of the survey
How do you rate your work ability with respect to the physical demands of your work?	1 – poor<br>2 – rather poor<br>3 – fair<br>4 – good<br>5 – very good	Low work ability in relation to physical job demand: score of less than 3 out of 5
How do you rate your work ability with respect to the mental demands of your work?	1 – poor<br>2 – rather poor<br>3 – fair<br>4 – good<br>5 – very good	Low work ability in relation to mental job demand: score of less than 3 out of 5
**Absenteeism**		
How many full working days have you been absent from work due to health reasons, for any cause, in the past 12 months (1 year)?	0 – never<br>1 – less than 10 days<br>2 – between 10 and 24 days<br>3 – between 25 and 99 days<br>4 – between 100 and 365 days<br>5 – not applicable	Long-term absenteeism was defined as 100 or more workdays missed during last year due to health-related issues

[1] Martus P, Jakob O, Rose U, Seibt R, Freude G. A comparative analysis of the Work Ability Index. Occup Med. 2010;60(7):517–24

Supplementary Table 3: Work role functioning questionnaire to evaluate work functioning[2]

Subscale	Items	Choice of answers and scores	Subscale score
**1. Work schedule demands**	1.1 Get going easily at the beginning of the workday<br>1.2 Start on your job as soon as you arrive at work<br>1.3 Do your work without stopping to take extra breaks or rests<br>1.4 Stick to a routine or schedule	0 – Difficult all the time = **100%**<br>1 –Difficult most of the time = **75%**<br>2 – Difficult half of the time = **50%**<br>3 –Difficult some of the time = **25%**<br>4 – Difficult none of the time = **0%**<br>NA – Not applicable	Subscale scores were calculated by averaging item scores and multiplying by 25 to generate scores from 0% to 100%. A subscale score was considered missing if 20% or more of the item responses were missing or not applicable.
**2. Work output demands**	2.1 Work fast enough<br>2.2 Finish work on time<br>2.3 Do your work without making mistakes<br>2.4 Satisfy the people who judge your work<br>2.5 Feel a sense of accomplishment in your work<br>2.6 Feel you have done what you are capable of doing	0 = **100%**<br>1 = **75%**<br>2 = **50%**<br>3 = **25%**<br>4 = **0%**<br>NA	Subscale scores were calculated by averaging item scores and multiplying by 25 to generate scores from 0% to 100%. A subscale score was considered missing if 20% or more of the item responses were missing or not applicable.
**3. Physical demands**	3.1 Lift, carry, or move objects at work weighing more than 10 pounds<br>3.2 Sit, stand, or stay in one position for longer than 15 minutes while working<br>3.3 Repeat the same motions over and over again while working<br>3.4 Bend, twist, or reach while working<br>3.5 Use hand-held tools or equipment	0 = **100%**<br>1 = **75%**<br>2 = **50%**<br>3 = **25%**<br>4 = **0%**<br>NA	Subscale scores were calculated by averaging item scores and multiplying by 25 to generate scores from 0% to 100%. A subscale score was considered missing if 20% or more of the item responses were missing or not applicable.
**4. Mental and social demands**	4.1 Keep your mind on your work<br>4.2 Do work carefully<br>4.3 Concentrate on your work<br>4.4 Work without losing your train of thought<br>4.5 Easily read or use your eyes when working<br>4.6 Speak with people in person, in meetings, or on the phone<br>4.7 Control your temper around people when working	0 = **100%**<br>1 = **75%**<br>2 = **50%**<br>3 = **25%**<br>4 = **0%**<br>NA	Subscale scores were calculated by averaging item scores and multiplying by 25 to generate scores from 0% to 100%. A subscale score was considered missing if 20% or more of the item responses were missing or not applicable.
**5. Flexibility demands**	5.1 Set priorities in my work<br>5.2 Handle changes in my work<br>5.3 Process incoming information, for example e-mails, in time<br>5.4 Perform multiple tasks at the same time<br>5.5 Be proactive, show initiative in my work	0 = **100%**<br>1 = **75%**<br>2 = **50%**<br>3 = **25%**<br>4 = **0%**<br>NA	Subscale scores were calculated by averaging item scores and multiplying by 25 to generate scores from 0% to 100%. A subscale score was considered missing if 20% or more of the item responses were missing or not applicable.
**Work functioning global score**			Mean score of valid items (score = 0-100%)

Abbreviations: NA, not applicable

[2] Abma FI, Bültmann U, Amick Iii BC, Arends I, Dorland HF, Flach PA, et al. The Work Role Functioning Questionnaire v2.0 Showed Consistent Factor Structure Across Six Working Samples. J Occup Rehabil. 2018;28(3):465–74

Supplementary Table 4: The Modified medical research council scale to evaluate dyspnea-associated impairment[3]

Items	Scores	Construction of indicators
I only get breathless with strenuous exercise	0	
I get short of breath when hurrying on level ground or walking up a slight hill	1	
On level ground, I walk slower than people of my age because of breathlessness, or I have to stop for breath when walking at my own pace on the level	2	Dyspnea-associated impairment was defined as a score greater than 1 out of 4
I stop for breath after walking about 100 yards or after a few minutes on level ground	3	
I am too breathless to leave the house, or I am breathless when dressing/undressing	4	

Note: Dyspnea-associated impairment was evaluated before the pandemic and at the time of the survey.

[3] Mahler DA, Wells CK. Evaluation of Clinical Methods for Rating Dyspnea. Chest. 1988;93(3):580–6

Supplementary Table 5: The Kessler scale K6 to evaluate the psychological distress[4]

Items and questions	Choice of answers and score for each answer	Construction of indicators
Psychological distress		
Over the last month, how often did you feel...		
A. nervous?	0: Never; 1: Rarely; 2: Sometimes; 3: Most of the time; 4: All the time; 5: Don't know; 6: Don't answer	Psychological distress.<br>Don't know and don't answer were considered as missing.<br>Sum of responses provided to questions A through F (0-4 points per question).<br>High distress = score ≥ 7<br>Very high distress = score ≥ 13
B. desperate?	0: Never; 1: Rarely; 2: Sometimes; 3: Most of the time; 4: All the time; 5: Don't know; 6: Don't answer	
C. agitated or unable to stay put?	0: Never; 1: Rarely; 2: Sometimes; 3: Most of the time; 4: All the time; 5: Don't know; 6: Don't answer	
D. so depressed that nothing could make you smile?	0: Never; 1: Rarely; 2: Sometimes; 3: Most of the time; 4: All the time; 5: Don't know; 6: Don't answer	
E. that everything was an effort (so much tired that everything requires an effort)?	0: Never; 1: Rarely; 2: Sometimes; 3: Most of the time; 4: All the time; 5: Don't know; 6: Don't answer	
F. worthless?	0: Never; 1: Rarely; 2: Sometimes; 3: Most of the time; 4: All the time; 5: Don't know; 6: Don't answer	
Link with the long COVID: *Do you believe that the feelings of last month are related to your work, personal life or/and the persisting symptoms post-COVID-19?*		Long COVID-related distress

4 Furukawa TA, Kessler RC, Slade T, Andrews G. The performance of the K6 and K10 screening scales for psychological distress in the Australian National Survey of Mental Health and Well-Being. Psychol Med. 2003;33(2):357–62

Supplementary Table 6: Prevalence differences and prevalence ratios of low work-related capacity, dyspnea-associated impairment, and high psychological distress among healthcare workers with long COVID

Outcome and subpopulation	N	Prevalence of each outcome		Unadjusted prevalence difference (95% CI) (%)	Adjusted[a] prevalence difference (95% CI) (%)	Unadjusted prevalence ratio (95% CI)	Adjusted[a] prevalence ratio (95% CI)
		Long COVID cases	Covid controls				
**Low work ability[b]**							
≥1 comorbidities	3276	20.5%	4.7%	15.8 (12.6-19.0)	14.9 (11.6-18.2)	4.4 (3.5-5.5)	4.2 (3.3-5.3)
CCVD	484	24.5%	3.9%	20.6 (11.9-29.3)	20.0 (10.6-29.5)	6.3 (3.4-11.6)	6.1 (3.2-11.7)
CRD	1051	22.9%	3.9%	19.0 (13.2-24.7)	17.9 (11.6-24.3)	5.8 (3.8-8.8)	5.7 (3.7-8.7)
Obesity	674	21.0%	5.5%	15.5 (8.6-22.5)	14.9 (7.4-22.5)	3.8 (2.4-6.2)	3.9 (2.5-6.4)
History of depression	1745	17.7%	6.2%	11.6 (7.4-15.7)	10.4 (6.2-14.6)	2.9 (2.1-3.9)	2.6 (1.9-3.6)
No comorbidity[c]	7610	13.1%	2.8%	10.3 (8.2-12.5)	10.0 (7.8-12.2)	4.7 (3.8-5.8)	4.5 (3.6-5.6)
**Low work functioning[b]**							
≥1 comorbidities	3028	51.9%	24.7%	27.2 (22.9-31.6)	26.7 (22.1-31.3)	2.1 (1.9-2.3)	2.1 (1.9-2.3)
CCVD	441	50.0%	21.0%	29.0 (17.5-40.5)	24.6 (11.7-37.4)	2.4 (1.8-3.2)	2.4 (1.8-3.2)
CRD	972	51.0%	23.5%	27.5 (19.9-35.1)	29.2 (20.7-37.8)	2.2 (1.8-2.6)	2.2 (1.8-2.6)
Obesity	614	53.0%	24.1%	29.0 (19.6-38.3)	30.7 (20.6-40.8)	2.2 (1.8-2.8)	2.3 (1.8-2.9)
History of depression	1621	55.2%	28.2%	27.0 (21.1-33.0)	26.3 (20.2-32.5)	1.9 (1.7-2.2)	1.9 (1.7-2.2)
No comorbidity[c]	7211	42.8%	21.6%	21.2 (17.8-24.6)	21.0 (17.5-24.5)	1.9 (1.8-2.2)	1.9 (1.8-2.1)
**Long-term absenteeism[b]**							
≥1 comorbidities	3528	17.3%	8.1%	9.2 (6.2-12.2)	7.9 (4.9-10.9)	2.1 (1.7-2.6)	1.9 (1.6-2.4)
CCVD	551	14.0%	7.2%	6.8 (-0.2-13.8)	6.4 (-1.1-14.0)	1.9 (1.1-3.5)	1.8 (0.9-3.4)
CRD	1142	19.6%	7.6%	12.0 (6.8-17.3)	10.8 (4.9-16.7)	2.6 (1.8-3.6)	2.3 (1.6-3.3)
Obesity	715	13.2%	9.0%	4.2 (-1.7-10.1)	2.9 (-3.0-8.9)	1.5 (0.9-2.4)	1.4 (0.9-2.3)
History of depression	1860	19.4%	10.2%	9.2 (5.0-13.4)	8.4 (4.1-12.8)	1.9 (1.5-2.4)	1.8 (1.4-2.3)
No comorbidity[c]	8040	11.1%	3.1%	8.0 (6.1-10.0)	7.5 (5.5-9.5)	3.6 (2.9-4.5)	3.4 (2.7-4.2)
**Dyspnea-associated impairment[b]**							
≥1 comorbidities	3754	30.9%	6.5%	24.5 (21.1-27.9)	22.5 (19.0-26.0)	4.8 (4.0-5.7)	4.3 (3.6-5.2)
CCVD	608	31.1%	7.2%	23.9 (15.3-32.6)	23.9 (14.2-33.6)	4.3 (2.9-6.6)	4.3 (2.7-6.7)
CRD	1204	33.1%	7.7%	25.4 (19.4-31.4)	21.2 (14.8-27.5)	4.3 (3.2-5.7)	3.6 (2.7-4.9)
Obesity	756	43.1%	9.7%	33.4 (25.4-41.4)	35.3 (26.4-44.2)	4.4 (3.3-5.9)	4.6 (3.4-6.3)
History of depression	1973	29.6%	6.9%	22.6 (18.0-27.2)	20.2 (15.6-24.8)	4.2 (3.4-5.4)	3.8 (3.0-4.7)

No comorbidity[c]	8439	16.4%	2.3%	14.1 (11.9-16.3)	13.0 (10.8-15.2)	7.1 (5.8-8.6)	6.5 (5.3-8.0)
**High psychological distress[b]**							
≥1 comorbidities	3585	69.1%	44.6%	25.2 (21.3-29.0)	24.4 (20.4-28.4)	1.6 (1.5-1.7)	1.6 (1.5-1.7)
CCVD	586	64.3%	34.8%	29.5 (19.8-39.3)	27.4 (16.4-38.4)	1.9 (1.5-2.2)	1.7 (1.5-2.1)
CRD	1150	68.9%	41.6%	27.2 (20.6-33.9)	27.4 (20.1-34.6)	1.7 (1.5-1.9)	1.6 (1.4-1.8)
Obesity	713	72.8%	46.9%	25.9 (17.7-34.1)	25.2 (16.2-34.2)	1.6 (1.4-1.8)	1.5 (1.3-1.7)
History of depression	1882	75.5%	51.2%	24.2 (19.2-29.2)	23.5 (18.3-28.7)	1.5 (1.4-1.6)	1.5 (1.4-1.6)
No comorbidity[c]	8088	62.9%	34.9%	28.1 (25.0-31.3)	27.0 (23.7-30.3)	1.8 (1.7-1.9)	1.8 (1.7-1.9)

[a] Adjusted for age, sex, occupation, workplace, number of comorbidities, social deprivation index, and material deprivation index.

[b]Low work ability out of 10, score <6; low work functioning out of 100, score ≤ 75; long-term absenteeism, ≥ 100 working days due to health issues; dyspnea-associated impairment, score > 1; high psychological distress, score ≥ 7.

[c] Comorbidities assessed to define “no comorbidity”: Acquired immunodeficiency syndrome/ Human immunodeficiency virus, alcohol abuse, anemia, cancer, cardiovascular disease, cerebrovascular disease, coagulopathy, dementia, depression, drug abuse, fluid and electrolyte disorders, hepatic disease, hypertension, hypothyroidism, immune system problems, neurological disorders, obesity, paralysis, peripheral vascular disorders, psychosis, pulmonary circulation disease, renal disease, rheumatoid arthritis, ulcer disease, weight loss, chronic pulmonary disease and respiratory disease.

Abbreviations: CI, confidence interval; CCVD, chronic cardiovascular disease; CRD, chronic respiratory disease.

Supplementary Table 7: Prevalence differences and prevalence ratios of low work-related capacity, dyspnea-associated impairment, and high psychological distress among healthcare workers with moderate or severe long COVID

Outcome and subpopulation based on comorbidities	N	Prevalence of each outcome		Unadjusted prevalence difference (95% CI) (%)	Adjusted[a] prevalence difference (95% CI) (%)	Unadjusted prevalence ratio (95% CI)	Adjusted[a] prevalence ratio (95% CI)
		Long COVID cases	COVID controls				
**Low work ability[b]**							
≥1 comorbidities	3143	25.2%	4.7%	20.5 (16.7-24.4)	20.1 (16.0-24.2)	5.4 (4.3-6.7)	5.1 (4.0-6.5)
CCVD	461	30.7%	3.9%	26.8 (16.2-37.4)	25.8 (13.9-37.6)	7.9 (4.3-14.4)	7.1 (3.8-13.4)
CRD	1007	28.2%	3.9%	24.3 (17.4-31.2)	23.7 (15.7-31.7)	7.2 (4.8-10.8)	7.0 (4.6-10.7)
Obesity	649	25.4%	5.5%	20.0 (11.9-28.1)	19.2 (10.3-28.2)	4.7 (2.9-7.5)	4.6 (2.9-7.5)
History of depression	1676	21.4%	6.2%	15.2 (10.2-20.1)	14.2 (9.1-19.3)	3.5 (2.6-4.7)	3.2 (2.3 -4.3)
No comorbidity[c]	7323	16.7%	2.8%	13.9 (11.0-16.8)	13.7 (10.7-16.7)	5.9 (4.8-7.5)	5.7 (4.5-7.2)
**Low work functioning[b]**							
≥1 comorbidities	2901	57.6%	24.7%	32.9 (28.0-37.7)	32.9 (27.7-38.1)	2.3 (2.1-2.6)	2.3 (2.1-2.6)
CCVD	419	50.0%	21.0%	29.0 (15.9-42.1)	22.6 (7.6-37.6)	2.4 (1.7-3.3)	2.3 (1.6-3.2)
CRD	931	56.1%	23.5%	32.5 (24.2-40.9)	35.1 (25.5-44.6)	2.4 (1.9-2.9)	2.4 (1.9-2.9)
Obesity	590	57.4%	24.1%	33.3 (23.3-43.4)	33.8 (22.6-45.0)	2.4 (1.9-2.9)	2.5 (1.9-3.1)
History of depression	1553	61.5%	28.2%	33.4 (27.0-39.8)	34.2 (27.4-41.0)	2.2 (1.9-2.5)	2.2 (1.9-2.5)
No comorbidity[c]	6928	50.0%	21.6%	28.4 (24.3-32.5)	28.6 (24.3-33.0)	2.3 (2.1-2.5)	2.2 (2.0-2.4)
**Long-term absenteeism[b]**							
≥1 comorbidities	3390	19.9%	8.1%	11.8 (8.4-15.3)	10.1 (6.6-13.6)	2.5 (2.0-3.0)	2.2 (1.8-2.7)
CCVD	527	18.1%	7.2%	10.9 (2.2-19.5)	10.2 (0.7-19.6)	2.5 (1.4-4.4)	2.3 (1.2-4.2)
CRD	1096	23.1%	7.6%	15.5 (9.4-21.6)	12.8 (6.1-19.5)	3.1 (2.2-4.3)	2.6 (1.8-3.8)
Obesity	690	15.9%	9.0%	6.8 (0.0-13.6)	4.4 (-2.2-10.9)	1.8 (1.1-2.8)	1.6 (0.9-2.7)
History of depression	1785	21.2%	10.2%	11.0 (6.2-15.8)	9.5 (4.6-14.4)	2.1 (1.6-2.7)	1.9 (1.5-2.5)
No comorbidity[c]	7735	13.8%	3.1%	10.6 (8.1-13.2)	10.1 (7.4-12.7)	4.5 (3.6-5.6)	4.1 (3.2-5.2)
**Dyspnea-associated impairment[b]**							
≥1 comorbidities	3608	35.7%	6.5%	29.2 (25.2-33.1)	27.0 (22.9-31.1)	5.5 (4.6-6.5)	4.9 (4.1-5.9)
CCVD	581	35.9%	7.2%	28.7 (18.7-38.8)	28.8 (17.2-40.5)	5.0 (3.3-7.6)	4.9 (3.1-7.8)
CRD	1157	38.6%	7.7%	30.9 (24.1-37.7)	25.4 (17.8-32.9)	5.0 (3.8-6.6)	4.2 (3.1-5.6)
Obesity	731	47.4%	9.7%	37.7 (28.9-46.4)	40.0 (30.3-49.8)	4.9 (3.6-6.6)	4.9 (3.6-6.7)
History of depression	1893	34.0%	6.9%	27.1 (21.8-32.3)	24.5 (19.1-29.9)	4.9 (3.9-6.2)	4.4 (3.4-5.6)

No comorbidity[c]	8120	21.8%	2.3%	19.5 (16.5-22.4)	17.8 (14.8-20.8)	9.4 (7.7-11.5)	8.4 (6.9-10.4)
**High psychological Distress[b]**							
≥1 comorbidities	3441	74.7%	44.6%	30.2 (26.2-34.2)	29.5 (25.2-33.8)	1.7 (1.6-1.8)	1.7 (1.6-1.8)
CCVD	559	68.2%	34.8%	33.4 (22.7-44.0)	32.4 (20.1-44.8)	1.9 (1.6-2.4)	1.9 (1.5-2.3)
CRD	1104	71.7%	41.6%	30.1 (23.1-37.2)	30.3 (22.5-38.0)	1.7 (1.5-1.9)	1.7 (1.5-1.9)
Obesity	689	75.6%	46.9%	28.6 (20.1-37.2)	26.4 (16.5-36.4)	1.6 (1.4-1.8)	1.6 (1.4-1.8)
History of depression	1803	81.1%	51.4%	29.9 (24.9-35.0)	30.2 (24.9-35.4)	1.6 (1.5-1.7)	1.6 (1.5-1.7)
No comorbidity[c]	7782	69.5%	34.9%	34.6 (31.0-38.2)	33.7 (29.9-37.6)	1.9 (1.9-2.1)	1.9 (1.8-2.1)

[a] Adjusted for age, sex, occupation, workplace, number of comorbidities, social deprivation index, and material deprivation index.

[b] Low work ability out of 10, score <6; low work functioning out of 100, WRFQ score ≤ 75; long-term absenteeism, ≥ 100 working days due to health issues; dyspnea-associated impairment, score > 1; high psychological distress, score ≥ 7.

[c] Comorbidities assessed to define “no comorbidity”: Acquired immunodeficiency syndrome/ Human immunodeficiency virus, alcohol abuse, anemia, cancer, cardiovascular disease, cerebrovascular disease, coagulopathy, dementia, depression, drug abuse, fluid and electrolyte disorders, hepatic disease, hypertension, hypothyroidism, immune system problems, neurological disorders, obesity, paralysis, peripheral vascular disorders, psychosis, pulmonary circulation disease, renal disease, rheumatoid arthritis, ulcer disease, weight loss, chronic pulmonary disease and respiratory disease.

Abbreviations: WRFQ, Work Role Functioning Questionnaire; CI, Confidence interval; CCVD, chronic cardiovascular disease; CRD, chronic respiratory disease.

Supplementary Table 8: Prevalence differences and prevalence ratios of low work ability, overall and in relation to physical and mental job demands among healthcare workers with long COVID

Outcome and subpopulation based on comorbidities	N	Prevalence of each outcome		Unadjusted prevalence difference (95% CI) (%)	Adjusted[a] prevalence difference (95% CI) (%)	Unadjusted prevalence ratio (95% CI)	Adjusted[a] prevalence ratio (95% CI)
		Long COVID cases	COVID controls				
**Overall low work ability[b]**							
≥1 comorbidities	3276	20.5%	4.7%	15.8 (12.6-19.0)	14.9 (11.6-18.2)	4.4 (3.5-5.5)	4.2 (3.3-5.3)
CCVD	484	24.5%	3.9%	20.6 (11.9-29.3)	20.0 (10.6-29.5)	6.3 (3.4-11.6)	6.1 (3.2-11.7)
CRD	1051	22.9%	3.9%	19.0 (13.2-24.7)	17.9 (11.6-24.3)	5.8 (3.8-8.8)	5.7 (3.7-8.7)
Obesity	674	21.0%	5.5%	15.5 (8.6-22.5)	14.9 (7.4-22.5)	3.8 (2.4-6.2)	3.9 (2.5-6.4)
History of depression	1745	17.7%	6.2%	11.6 (7.4-15.7)	10.4 (6.2-14.6)	2.9 (2.1-3.9)	2.6 (1.9-3.6)
No comorbidity[c]	7610	13.1%	2.8%	10.3 (8.2-12.5)	10.0 (7.8-12.2)	4.7 (3.8-5.8)	4.5 (3.6-5.6)
**Low work ability in relation to physical job demands[b]**							
≥1 comorbidities	3276	25.2%	4.7%	20.4 (17.0-23.9)	18.5 (15.1-21.9)	5.3 (4.3-6.6)	4.8 (3.9-6.0)
CCVD	484	26.5%	4.7%	21.9 (12.9-30.9)	21.0 (11.0-31.0)	5.7 (3.3-9.9)	5.1 (2.8-9.4)
CRD	1051	28.9%	5.4%	23.6 (17.3-29.9)	20.0 (13.5-26.6)	5.4 (3.8-7.7)	4.8 (3.3-6.9)
Obesity	674	27.3%	6.0%	21.3 (13.7-28.8)	20.8 (12.6-29.1)	4.5 (2.9-6.9)	4.8 (3.1-7.4)
History of depression	1745	21.4%	5.4%	16.1 (11.6-20.5)	13.8 (9.4-18.1)	3.9 (2.9-5.4)	3.4 (2.5-4.7)
No comorbidity[c]	7610	16.6%	2.8%	13.7 (11.3-16.1)	13.1 (10.6-15.5)	5.8 (4.7-7.1)	5.4 (4.4-6.6)
**Low work ability in relation to mental job demands[b]**							
≥1 comorbidities	3276	21.6%	7.3%	14.3 (10.9-17.6)	13.9 (10.4-17.3)	2.9 (2.4-3.6)	2.9 (2.3-3.5)
CCVD	484	20.4%	6.2%	14.2 (5.9-22.5)	15.5 (5.9-25.0)	3.3 (1.9-5.7)	3.2 (1.8-5.7)
CRD	1051	21.5%	6.3%	15.2 (9.4-20.9)	14.6 (8.4-20.9)	3.4 (2.4-4.9)	3.5 (2.5-5.1)
Obesity	674	26.6%	8.5%	18.1 (10.5-25.7)	18.7 (10.2-27.2)	3.1 (2.1-4.6)	3.2 (2.1-4.7)
History of depression	1745	21.1%	8.8%	12.3 (7.8-16.9)	11.7 (7.0-16.3)	2.4 (1.8-3.1)	2.3 (1.8-2.9)
No comorbidity[c]	7610	17.5%	4.9%	12.6 (10.2-15.1)	12.5 (9.9-15.0)	3.6 (3.0-4.3)	3.5 (2.9-4.1)

[a] Adjusted for age, sex, occupation, workplace, number of comorbidities, social deprivation index, and material deprivation index.

[b] Overall low work ability out of 10, score <6; low work ability in relation to physical and mental demand out of 5, score <3

[c] Comorbidities assessed to define “no comorbidity”: Acquired immunodeficiency syndrome/ Human immunodeficiency virus, alcohol abuse, anemia, cancer, cardiovascular disease, cerebrovascular disease, coagulopathy, dementia, depression, drug abuse, fluid and electrolyte disorders, hepatic disease, hypertension, hypothyroidism, immune system problems, neurological disorders, obesity, paralysis, peripheral vascular disorders, psychosis, pulmonary circulation disease, renal disease, rheumatoid arthritis, ulcer disease, weight loss, chronic pulmonary disease and respiratory disease.

Abbreviations: CI, Confidence interval; CCVD, chronic cardiovascular disease; CRD, chronic respiratory disease.

Supplementary Table 9: Prevalence differences and prevalence ratios of the five subscales of low work functioning among healthcare workers with long COVID

Outcome and subpopulation based on comorbidities	N	Prevalence of each outcome		Unadjusted prevalence difference (95% CI) (%)	Adjusted[a] prevalence difference (95% CI) (%)	Unadjusted prevalence ratio (95% CI)	Adjusted[a] prevalence ratio (95% CI)
		Long COVID cases	COVID controls				
**Low work schedule demand[b]**							
≥1 comorbidities	1176	85.6%	71.9%	13.7 (8.7-18.6)	12.8 (7.7-17.9)	1.2 (1.1-1.3)	1.2 (1.1-1.2)
CCVD	154	82.9%	69.9%	13.0 (-1.3-27.3)	15.0 (-0.7-30.7)	1.2 (0.9-1.4)	1.2 (0.9-1.5)
CRD	365	88.1%	69.7%	18.4 (10.0-26.8)	17.5 (6.9-28.0)	1.3 (1.1-1.4)	1.3 (1.1-1.4)
Obesity	229	85.7%	74.8%	10.9 (0.3-21.5)	12.7 (2.7-22.7)	1.2 (1.0-1.3)	1.1 (0.9-1.3)
History of depression	702	86.1%	73.5%	12.6 (6.2-19.0)	11.8 (5.1-18.5)	1.2 (1.1-1.3)	1.2 (1.1-1.2)
No comorbidity[c]	2613	79.3%	68.1%	11.3 (7.1-15.5)	9.9 (5.5-14.4)	1.2 (1.1-1.2)	1.2 (1.1-1.2)
**Low work output demand[b]**							
≥1 comorbidities	2777	52.6%	30.4%	22.2 (17.5-26.9)	22.2 (17.3-27.1)	1.7 (1.6-1.9)	1.7 (1.6-1.9)
CCVD	397	59.7%	26.2%	33.6 (21.3-45.9)	28.4 (14.5-42.3)	2.3 (1.8-2.9)	2.3 (1.8-2.9)
CRD	894	53.1%	30.1%	23.0 (14.9-31.1)	25.3 (16.1-34.4)	1.8 (1.5-2.1)	1.8 (1.5-2.2)
Obesity	572	54.2%	30.3%	23.9 (14.0-33.7)	24.0 (13.3-34.7)	1.8 (1.4-2.2)	1.8 (1.5-2.3)
History of depression	1483	53.9%	33.5%	20.4 (14.0-26.9)	19.1 (12.3-25.8)	1.6 (1.4-1.8)	1.6 (1.4-1.8)
No comorbidity[c]	6677	47.3%	26.8%	20.5 (16.8-24.2)	20.5 (16.8-24.3)	1.8 (1.6-1.9)	1.7 (1.6-1.9)
**Low physical demand[b]**							
≥1 comorbidities	1480	55.9%	28.2%	27.8 (21.5-34.0)	25.8% (19.3%-32.4%)	1.9 (1.7-2.3)	1.9 (1.6-2.1)
CCVD	217	52.4%	26.9%	25.5 (9.1-42.0)	27.8% (8.8%-46.7%)	1.9 (1.3-2.9)	2.2 (1.5-3.2)
CRD	485	55.6%	26.3%	29.3 (18,9-39.7%)	25.4% (13.7%-37.1%)	2.1 (1.7-2.7)	1.9 (1.5-2.4)
Obesity	325	59.2%	30.3%	28.8 (16.1-41.6)	28.7 (15.1-42.3)	1.9 (1.5-2.6)	1.9 (1.5-2.6)
History of depression	773	57.7%	30.6%	27.1 (18.5-35.8)	26.2 (17.2-35.2)	1.9 (1.6-2.3)	1.8 (1.5-2.1)
No comorbidity[c]	3661	47.4%	24.1%	23.4 (18.5-28.2)	22.1 (17.0-27.1)	1.9 (1.8-2.2)	1.8 (1.6-2.1)
**Low mental and social demand[b]**							
≥1 comorbidities	2901	50.6%	25.8%	24.9 (20.3-29.4)	24.8 (20.1-29.6)	1.9 (1.8-2.2)	1.9 (1.8-2.2)
CCVD	422	43.0%	21.9%	21.2 (9.4-32.9)	14.6 (2.1-27.0)	1.9 (1.4-2.7)	1.9 (1.4-2.8)
CRD	929	45.6%	25.4%	20.2 (12.3-28.1)	21.6 (12.8-30.3)	1.8 (1.5-2.2)	1.8 (1.5-2.2)
Obesity	588	54.9%	25.3%	29.6 (19.9-39.3)	29.8 (19.4-40.2)	2.2 (1.7-2.7)	2.2 (1.7-2.7)
History of depression	1557	54.4%	28.3%	26.1 (20.0-32.2)	26.5 (20.1-32.9)	1.9 (1.7-2.2)	1.9 (1.7-2.2)
No comorbidity[c]	6943	42.9%	22.7%	20.2 (16.7-23.7)	20.6 (16.9-24.2)	1.9 (1.7-2.1)	1.9 (1.7-2.0)

**Low flexibility demand**[b]							
≥1 comorbidities	2744	50.1%	31.0%	19.1 (14.4-23.8)	19.2 (14.4-24.1)	1.6 (1.5-1.8)	1.6 (1.5-1.8)
CCVD	395	43.6%	29.3%	14.3 (2.2-26.3)	7.9 (-5.0-20.9)	1.5 (1.1-2.0)	1.5 (1.1-1.9)
CRD	884	51.2%	30.1%	21.1 (12.8-29.3)	24.1 (14.9-33.3)	1.7 (1.4-2.0)	1.8 (1.5-2.1)
Obesity	558	52.5%	29.3%	23.2 (13.3-33.2)	23.7 (13.0-34.5)	1.8 (1.4-2.2)	1.8 (1.4-2.3)
History of depression	1471	51.2%	34.3%	16.9 (10.5-23.2)	16.9 (10.3-23.5)	1.5 (1.3-1.7)	1.5 (1.3-1.7)
No comorbidity[c]	6584	44.8%	28.6%	16.2 (12.5-19.8)	16.7 (13.0-20.5)	1.6 (1.4-1.7)	1.6 (1.4-1.7)

[a] Adjusted for age, sex, occupation, workplace, number of comorbidities, social deprivation index, and material deprivation index.

[b] Low work schedule demand, work output demand, physical demand, mental and social demand, flexibility demand, score ≤75.

[c] Comorbidities assessed to define "no comorbidity": Acquired immunodeficiency syndrome/ Human immunodeficiency virus, alcohol abuse, anemia, cancer, cardiovascular disease, cerebrovascular disease, coagulopathy, dementia, depression, drug abuse, fluid and electrolyte disorders, hepatic disease, hypertension, hypothyroidism, immune system problems, neurological disorders, obesity, paralysis, peripheral vascular disorders, psychosis, pulmonary circulation disease, renal disease, rheumatoid arthritis, ulcer disease, weight loss, chronic pulmonary disease and respiratory disease.

Abbreviations: CI, Confidence interval; CCVD, chronic cardiovascular disease; CRD, chronic respiratory disease.

Supplementary Table 10: Prevalence differences and prevalence ratios of very high psychological distress among healthcare workers with long COVID

Subpopulation based on comorbidities	N	Prevalence of very high psychological distress		Unadjusted prevalence difference (95% CI) (%)	Adjusted[a] prevalence difference (95% CI) (%)	Unadjusted prevalence ratio (95% CI)	Adjusted[a] prevalence ratio (95% CI)
		**Long COVID cases**	**COVID controls**				
≥1 comorbidities	**3585**	31.9%	12.0%	19.8 (16.2-23.5)	19.3 (15.5-23.1)	2.7 (2.3-3.1)	2.6 (2.3-3.1)
CCVD	**586**	24.4%	8.1%	16.3 (8.1-24.5)	14.5 (5.7-23.3)	3.0 (1.9-4.7)	3.0 (1.9-4.7)
CRD	**1150**	29.5%	11.4%	18.1 (12.1-24.2)	18.2 (11.4-25.1)	2.6 (1.9-3.4)	2.5 (1.9-3.2)
Obesity	**713**	37.1%	14.8%	22.3 (14.1-30.6)	23.4 (14.1-32.8)	2.5 (1.9-3.4)	2.5 (1.9-3.4)
History of depression	**1882**	35.7%	14.5%	21.1 (16.1-26.2)	20.3 (15.1-25.5)	2.5 (2.1-2.9)	2.1 (2.0-2.9)
No comorbidity[b]	**8088**	23.8%	8.6%	15.2 (12.5-17.9)	14.8 (12.0-17.5)	2.8 (2.4-3.2)	2.7 (2.3-3.0)

[a] Adjusted for age, sex, occupation, workplace, number of comorbidities, social deprivation index, and material deprivation index.

[b] Comorbidities assessed to define "no comorbidity": Acquired immunodeficiency syndrome/ Human immunodeficiency virus, alcohol abuse, anemia, cancer, cardiovascular disease, cerebrovascular disease, coagulopathy, dementia, depression, drug abuse, fluid and electrolyte disorders, hepatic disease, hypertension, hypothyroidism, immune system problems, neurological disorders, obesity, paralysis, peripheral vascular disorders, psychosis, pulmonary circulation disease, renal disease, rheumatoid arthritis, ulcer disease, weight loss, chronic pulmonary disease and respiratory disease.

Note: Very high psychological distress, score ≥ 13.

Abbreviations: CI, Confidence interval; CCVD, chronic cardiovascular disease; CRD, chronic respiratory disease.

Supplementary Figure 1: Prevalence of low work ability, low work functioning, long-term absenteeism, and dyspnea-associated impairment among healthcare workers without long COVID

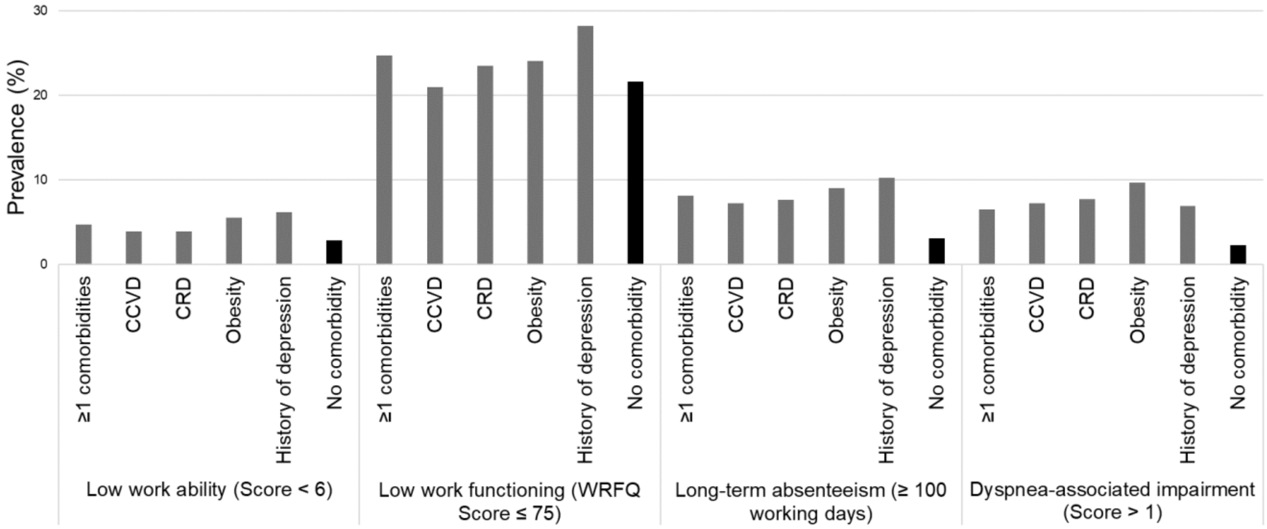

Note: Adjusted for age, sex, occupation, workplace, number of comorbidities, social deprivation index, and material deprivation index.

Abbreviations: CCVD, chronic cardiovascular disease; CRD, chronic respiratory disease; WRFQ, Work role functioning questionnaire; CI, confidence interval.

Supplementary Figure 2: Prevalence of self-reported low work ability and dyspnea-associated impairment of healthcare workers before the pandemic

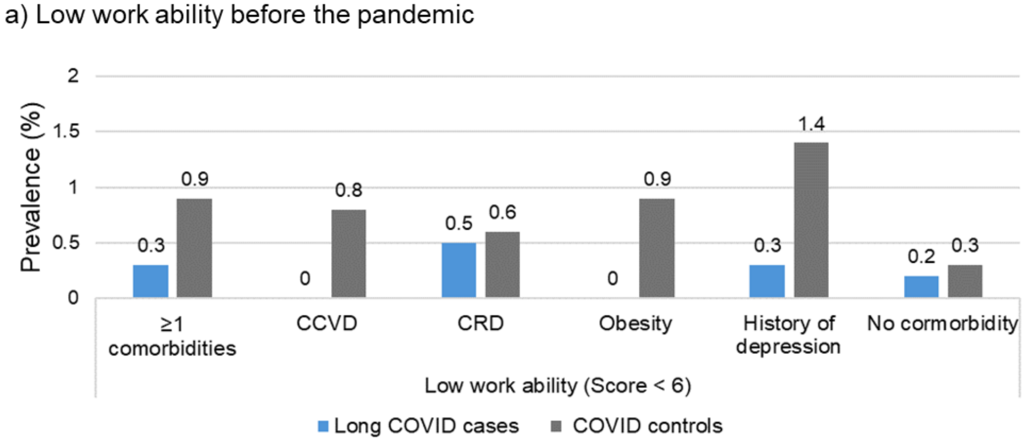

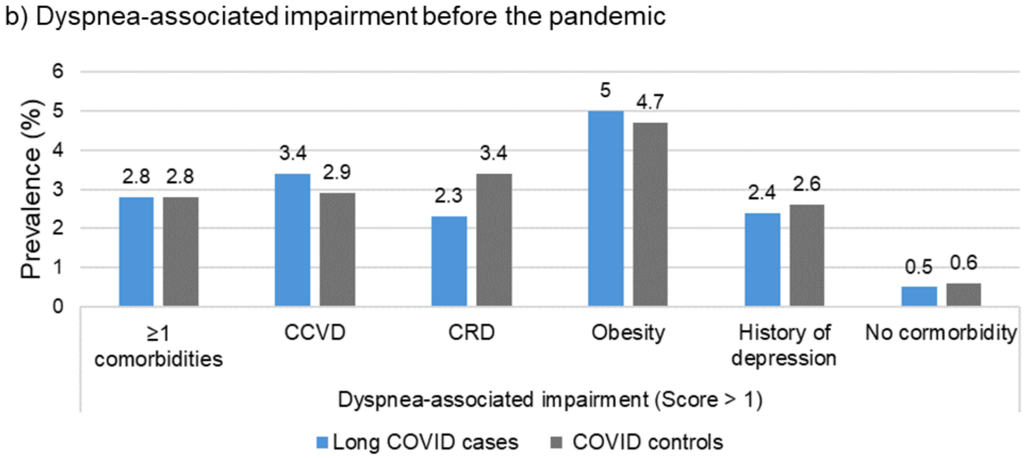

Note: Adjusted for age, sex, occupation, workplace, number of comorbidities, social deprivation index, and material deprivation index.

Abbreviations: CCVD, chronic cardiovascular disease; CRD, chronic respiratory disease; HCWs, healthcare workers.

Supplementary Figure 3: Prevalence of high psychological distress among healthcare workers at the time of the survey and its attribution to the post-COVID-19 symptoms

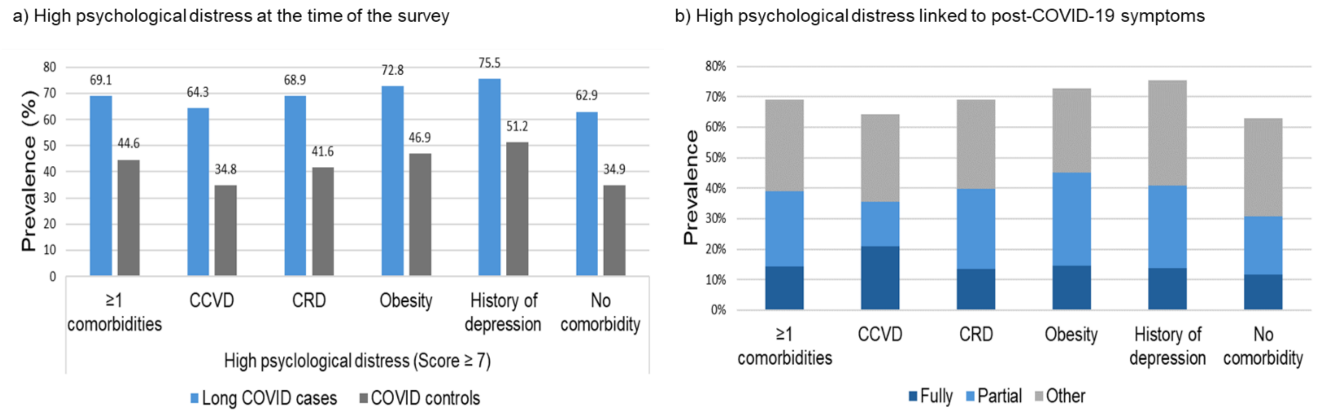

Adjusted for age, sex, occupation, workplace, number of comorbidities, social deprivation index, and material deprivation index.

Abbreviations: CCVD, chronic cardiovascular disease; CRD, chronic respiratory disease.
